# Laparoscopic treatment for an intrapancreatic accessory spleen: A case report

**DOI:** 10.3389/fonc.2022.972883

**Published:** 2022-10-05

**Authors:** Yihan Zhang, Guodong Shi, Lingdong Meng, Jing Wu, Qingqiao Hu, Dong Xv, Kai Zhang, Zipeng Lu, Junlii Wu, Kuirong Jiang

**Affiliations:** ^1^ Pancreas Center, the First Affiliated Hospital of Nanjing Medical University, Nanjing, China; ^2^ Pancreas Institute, Nanjing Medical University, Nanjing, China; ^3^ Department of Pathology, The First Affiliated Hospital of Nanjing Medical University, Nanjing, China; ^4^ Department of Nuclear Medicine, The First Affiliated Hospital of Nanjing Medical University, Nanjing, China

**Keywords:** intrapancreatic accessory spleen, pancreatic neuroendocrine tumor, imaging diagnosis, pathological diagnosis, surgery

## Abstract

Malignant pancreatic tumors have early metastasis, aggressive behavior and poor prognosis. Surgeons often need to judge whether a patient needs prompt surgery when a pancreatic lesion is found. The accessory spleen is a congenital developmental malformation rather than a tumor and does not require surgical resection. Here, we report a 47-year-old man who underwent routine gastroscopic examination, and a submucosal eminence of the duodenal bulb was detected. The patient was asymptomatic and laboratory tests were unremarkable. Duodenal neuroendocrine neoplasm (G2) was considered following endoscopic submucosal dissection (ESD). Further examination showed a lesion in the tail of the pancreas and multiple accessory spleens. The lesion in the tail of the pancreas was Ga-68 positive and was highly considered a pancreatic neuroendocrine tumor (pNET). Based on this clinical evidence, laparoscopic spleen-preserving distal pancreatectomy (Kimura) was performed. However, the results of the postoperative pathological diagnosis indicated an intrapancreatic accessory spleen (IPAS). Given the findings of this case, we should explore more accurate diagnostic methods for IPAS to avoid unnecessary surgery.

## Introduction

Accessory spleen (AS) is a congenital developmental malformation that mainly occurs in the splenic hilum (62.1%) and tail of the pancreas (5.5%) (1–3). Researchers have found that approximately 10% to 40% of patients have AS at autopsy (4). A large meta-analysis of patients with AS showed that those in Oceania had the highest incidence of AS (26.6%), followed by patients in North America (16.7%), Asia (14.1%) and Europe (12.2%) (5).

Advances in imaging diagnosis technology have helped identify an increasing number of people with pancreatic masses and, simultaneously, AS. ASs located in the pancreas are defined as intrapancreatic accessory spleens (IPASs). IPASs usually show no obvious symptoms and are often discovered in routine radiology examinations. There are no significant radiological differences between IPASs and pancreatic neuroendocrine tumors (pNETs), which may lead to a misdiagnosis. Here, we discuss methods for accurately diagnosing IPAS through a case encountered in our center.

## Case

A submucosal eminence of the duodenal bulb was discovered in a 47-year-old male patient after agreeing to undergo routine electronic gastroscopy in a local hospital. The eminence measured approximately 3*3 cm and presented with hyperemia on its surface. The patient had no chest tightness, asthma, bloating, abdominal pain or other symptoms. Ten days later, he was transferred to another hospital and underwent an abdominal enhanced CT scan as well as endoscopic submucosal dissection (ESD). The pathology results after ESD indicated that the mass was a neuroendocrine tumor (NET), the immunohistochemical report showed CgA (+), Syn (+), CD56 (+/-), SSTR2 (+), and Ki67 (4%+), and the mitotic count was 0-1/10 high-power fields (HPF). The mass was diagnosed as a NET-G2.

**Figure 1:**
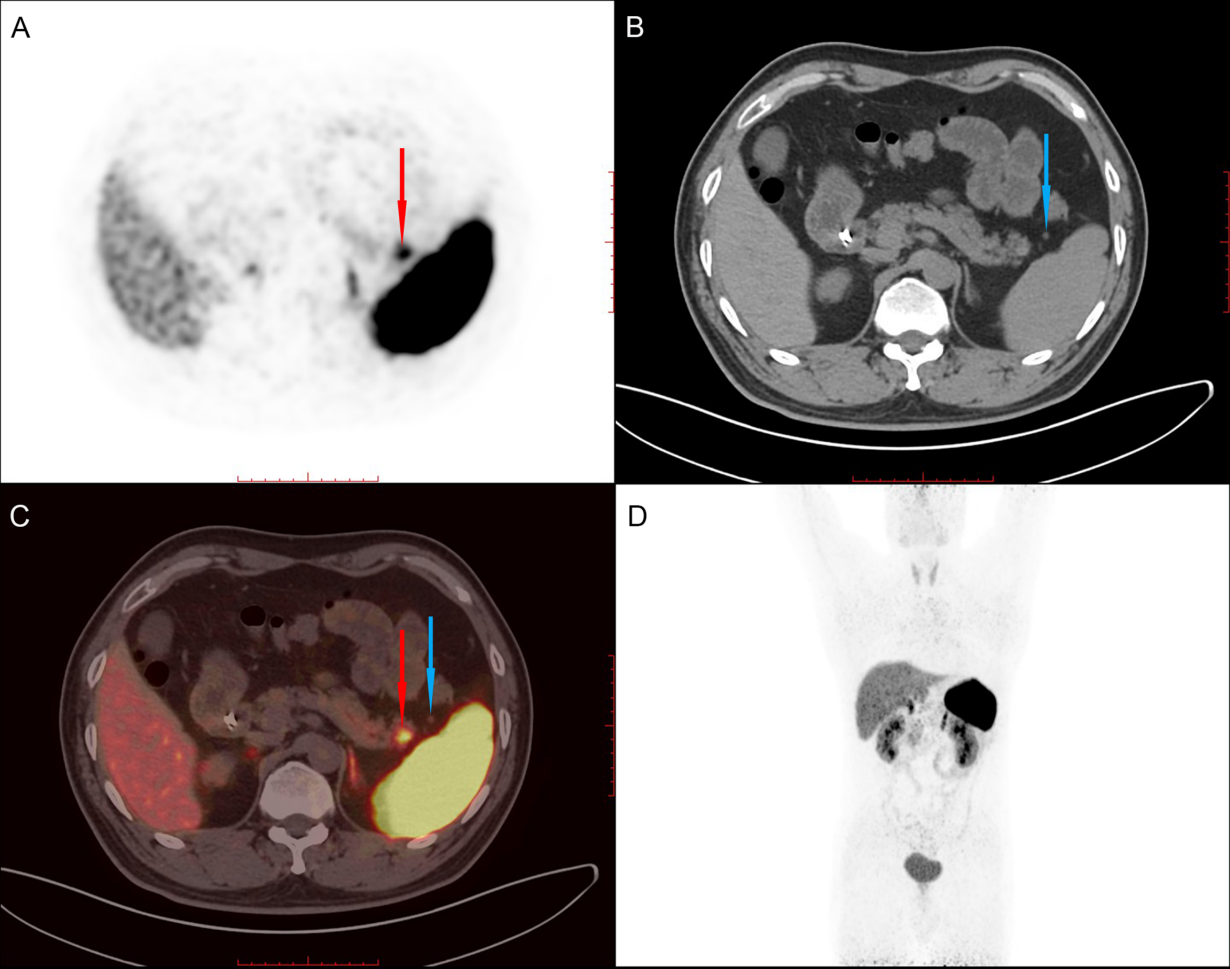
68Ga PET/CT shows a high-uptake nodule in the tail of the pancreas (red arrow) and an accessory spleen (blue arrow) with low uptake around the pancreas. **(A)**. PET image. **(B)**. CT image. **(C)** Fused PET/CT image. **(D)**. Whole-body PET image.

Given the malignant and metastatic potential of NETs and that the pathological grade of metastatic lesions may be higher than that of primary lesions, the patient underwent Ga-68 examination at a third hospital to evaluation whether other NETs were present in the abdomen and the rest of the body. Imaging revealed a high-uptake nodule in the tail of the pancreas and three accessory spleens with low uptake around the pancreas (**Figure 1** and **SFigures 1**, **2**)

**Figure 2:**
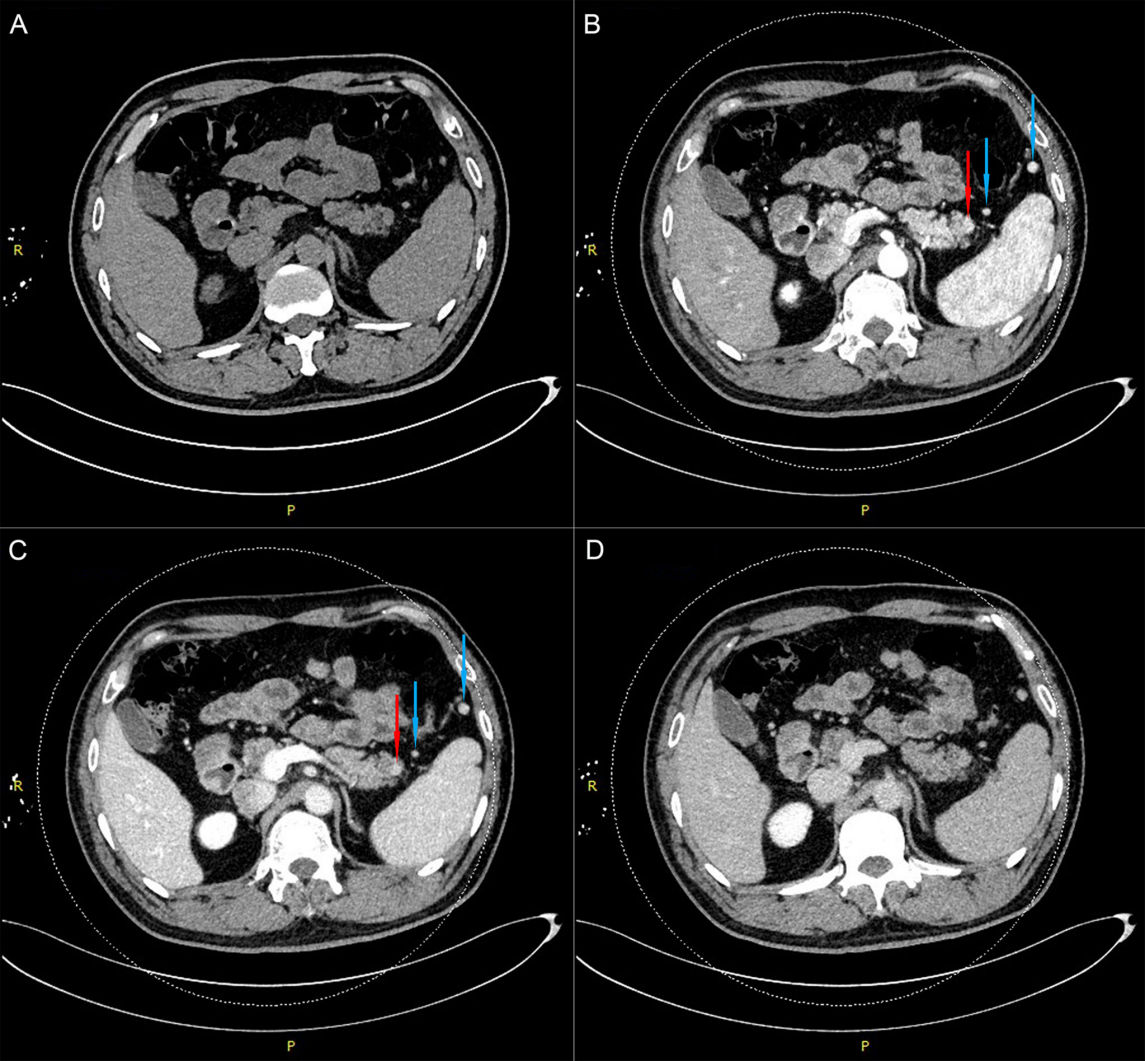
Dual-energy CT examination of the pancreas shows a nodular soft tissue density shadow in the tail of the pancreas (red arrow) and two accessory spleens near the pancreas and spleen (blue arrows). **(A)**. Plain-scan phase. **(B)**. Arterial phase. **(C)**. Venous phase. **(D)**. Delayed phase.

**Figure 3:**
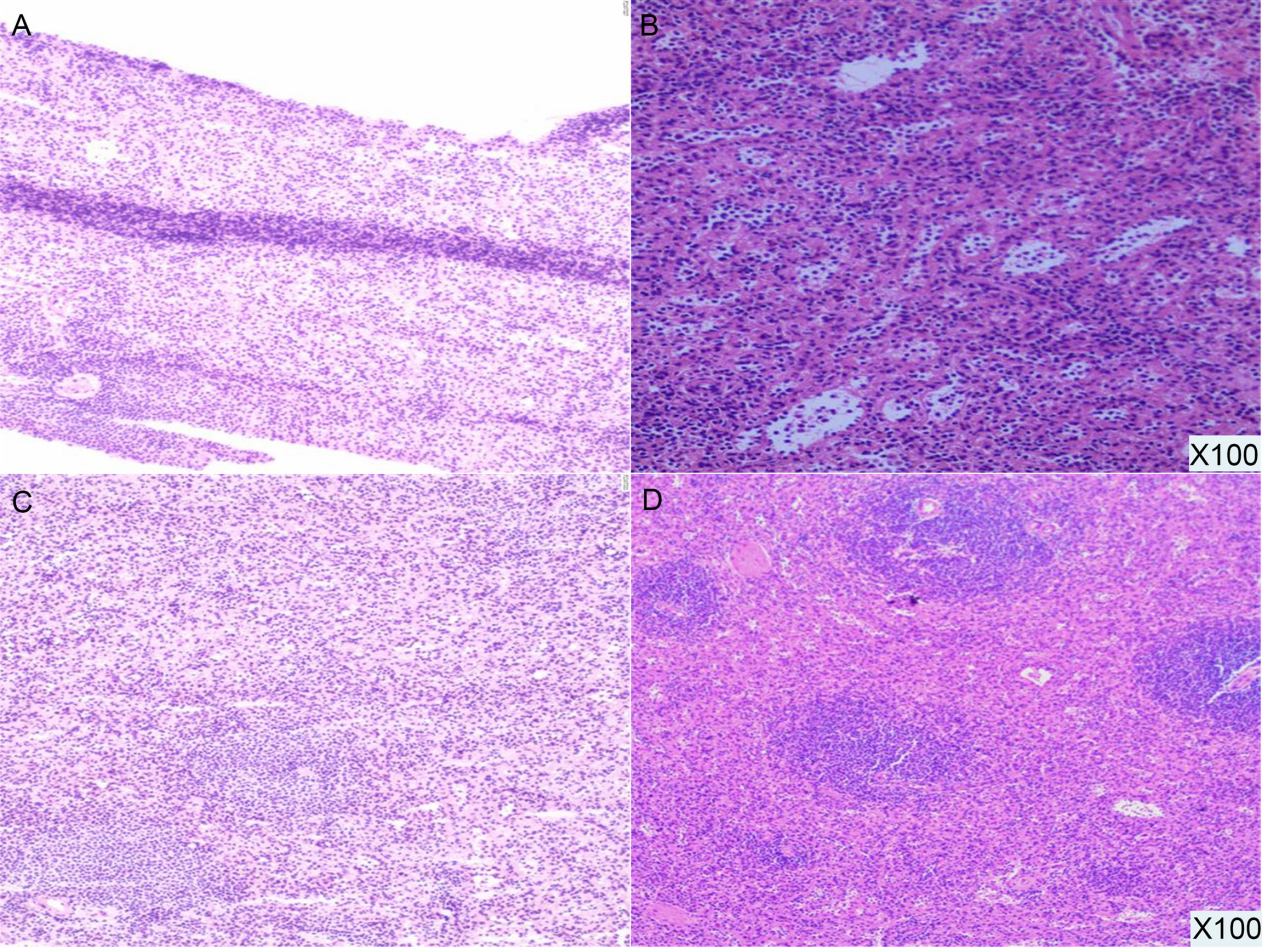
Pathological results (100X). The pancreatic tail lesion is rich in blood sinus structures, and lymphoid follicles and sinusoid-like structures can be seen.

For further treatment of the pancreatic tail lesion, the patient was transferred to our hospital. Neither his physical nor experimental examinations showed remarkable disorders. The patient’s serum tumor markers (CA19-9, CA125, alpha fetoprotein (AFP) and carcinoembryonic antigen (CEA)) were normal. After admission, endoscopic ultrasonography (EUS) was performed. We found a quasicircular, slightly hypoechoic lesion measuring approximately 10*8 mm. The lesion was located in the tail of the pancreas, close to the splenic hilus. To further clarify the nature of the lesion and uncover other lesions, we performed dual-energy CT (DECT) of the pancreas before continuing with the next treatment steps. From the CT scan, we observed a nodular soft tissue density shadow in the tail of the pancreas measuring approximately 10*11 mm. It was significantly enhanced in the arterial phase, weakly enhanced in the venous phase and had a slightly low density in the delayed phase. Additionally, there were multiple soft tissue density shadows around the spleen, which were considered small ASs (**Figure 2**, **SFigure 3**). Although the CT results showed that the lesion was similar to the ASs outside the spleen in density and enhancement characteristics, there was a great difference between these two kinds of lesions on PET/CT (high uptake/low uptake). Combined with the patient’s history of duodenal NET, we continued to highly consider the lesion to be a pancreatic NET (pNET). Based on the results above, the patient underwent laparoscopic spleen-preserving distal pancreatectomy (Kimura). The stapler technique for stump closure was performed after left pancreatectomy. This patient had no postoperative complication including a clinically relevant postoperative pancreatic fistula (CR-POPF) and was successfully discharged on postoperative day(POD) 10. The amylase level of drain fluid was high at 15569U/L and the drains were removed on POD 9, Based on the 2016 International Study Group (ISGPS) definition and grading of POPF, it belonged to a biochemical leak, but not a CR-POPF. Postoperative pathology showed pancreatic tissue, lymphoid follicles and blood sinus-like structures (**Figure 3**). The immunohistochemical results showed CD20 (+), CD3 (+), CK-pan (-), and Syn (-). The lesion was considered an IPAS rather than a pNET. After a postoperative follow-up of 12 months, the patient had no recurrence.

**Figure 4:**
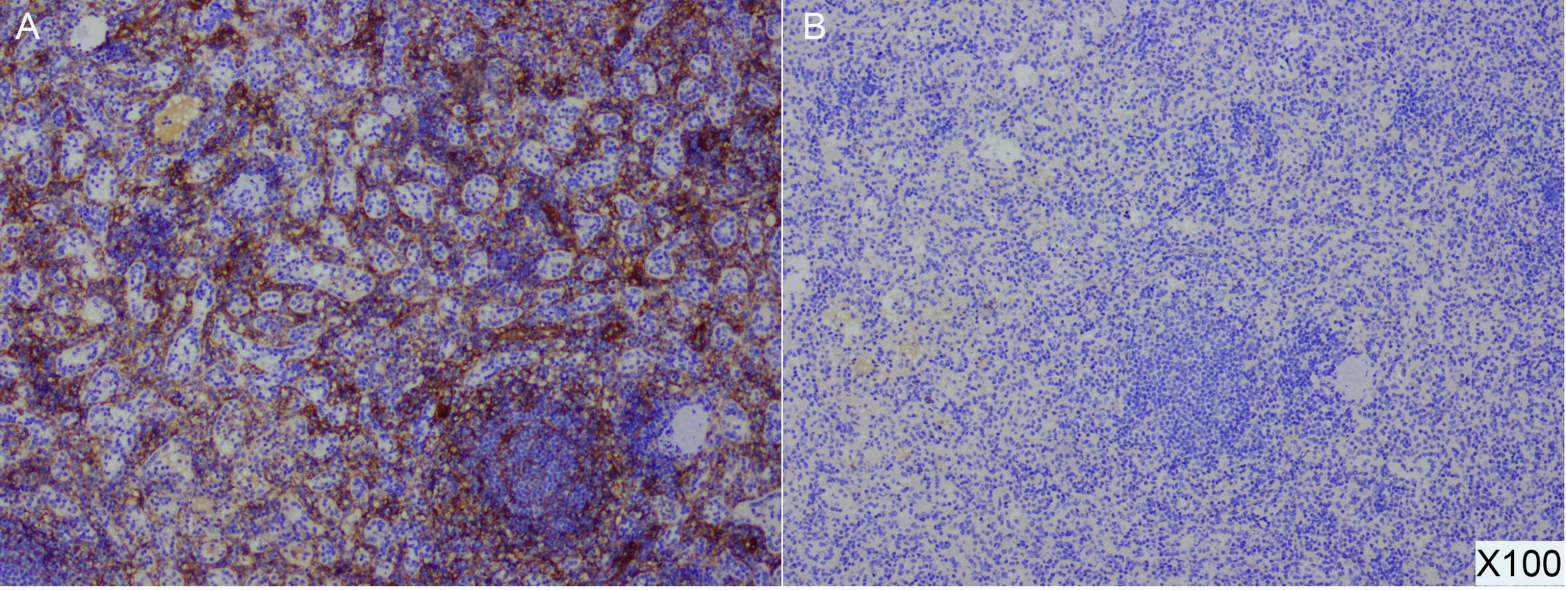
Postoperative pathological specimens and immunohistochemical results of IPAS demonstrate SSTR2(+) **(A)** and SSTR5 (-) **(B)** (100X).

The diagnosis of pNET by Ga-68 PET/CT mainly depends on the high expression of SSTR (mainly somatostatin receptor 2 (SSTR2) and 5 (SSTR5)). According to the earlier PET/CT results of this case, the lesion of the pancreatic tail was Ga-68 positive, indicating a highly likelihood of a misdiagnosis of NET. Therefore, we further performed additional immunohistochemical experiments. The SSTR results were SSTR2(+) and SSTR5(-) (**Figure 4**). In addition to SSTR, three other markers were tested, and the results were CD68(+), ERG(+), and CD8(+). Combined with hematoxylin and eosin (HE) sectioning and immunohistochemistry, this case was finally diagnosed as an intrapancreatic accessory spleen.

## Discussion

In this case, an IPAS was misdiagnosed as pNET, resulting in unnecessary surgery. However, the patient underwent relatively sufficient examinations before the operation including radiography, endoscopy and PET/CT imaging. The preoperative examination results did not suggest the possibility of a diagnosis of IPAS, which led to the implementation of unnecessary surgery. Given the results of this case, we attempted to determine the causes of the misdiagnosis and identify better differential diagnosis methods.

Usually, CT is the first choice in the diagnosis of pancreatic masses, as it can be helpful in determining the location of the mass, analyzing the relationship between masses and blood vessels, and preliminarily evaluating the tumor stage. In this case, the pancreatic tail mass was significantly enhanced in the arterial phase. Although the enhancement was weaker in the venous phase, it was still higher than that of the surrounding tissue. These hypervascular manifestations are consistent with the radiological characteristics of pNET. However, we ignored the fact that the mass matched the density of the spleen at all phases. This feature is an important difference between IPASs and pNETs. This characteristic change in density may be difficult to detect due to a lack of individual clinical experience or inaccurate CT phase capture. We consider that additional MRI may have helped to clarify the diagnosis. IPASs match the intensity of all sequences of the spleen on MRI. Although the intensity of both pNETs and IPASs shows a low T1 and high T2, pNETs can have a ring-like enhancement (6). However, pNETs with high fibrosis have no significant or delayed intensity changes between sequences, which makes it difficult to distinguish them from IPASs (7). Val-bernal et al. reported 4 cases of pancreatic tail lesions, all of which were diagnosed as pNETs by CT and MRI. However, three of them were pathologically confirmed as IPASs *via* endoscopic ultrasound-fine needle aspiration (EUS-FNA) biopsy (8). IPAS cannot be diagnosed accurately only through imaging examinations.

EUS is helpful for making diagnoses with less invasive trauma. In this case, the endosonographers used EUS and described that the lesion was quasicircular and slightly hypoechoic with a clear boundary. However, IPASs and pNETs have similar profiles on EUS, such as round and homogenous lesions with clear and regular boundaries, which means it is still difficult to make a diagnosis excluding pNET with this imaging modality (1). To improve the accuracy of the diagnosis, biopsy *via* EUS or ESD is often needed. At present, a variety of markers are used in the pathological differential diagnosis of IPAS and pNET, such as CK-pan, Vim (epithelial and neural markers), CgA, Syn, CD56 (NET diagnostic markers) and Ki-67 (grading markers). It is worth noting that CD8 has been reported to be more conducive in improving the accuracy of the differential diagnosis between IPAS and pNET (2). We believe that CD8 staining should be added on the basis of pNET-related immunohistochemistry.

The case serves as a reminder to consider combining various methods to help to make a more precise diagnosis. Some researchers have found that the use of PET/CT can enhance the accuracy of diagnosis. 68-Ga PET/CT has an important role in the differential diagnosis of pNETs from other pancreatic lesions, and it could be used as an exclusive diagnostic method to distinguish pNETs from IPASs. However, because of the physiological radioisotope uptake of 68-Ga in splenic tissue, false positive results in Ga-68 PET/CT have been reported, leading to misdiagnoses of IPASs as pNETs (9, 10). Similarly, in our case, Ga-68 PET/CT was performed but was unable to yield a correct diagnosis. Liberini (11) et al. reported on a fully convergent iterative image reconstruction algorithm with β‐values of 1000 (BSREM1000) and a data‐driven gating (DDG) technique for correcting ^68^Ga-Dotatate PET/CT data and more effectively distinguishing pNETs from IPASs.

In addition to calibrating ^68^Ga PET/CT data, we also focused on finding another method. Tc-99m heat-denatured red blood cell single photon emission computed tomography has recently been shown to be a better choice for differentiating pNETs from IPASs (12). The use of this technique for diagnosing the spleen dates back to the 1980s, when Dworkin et al. (13) identified the presence and size of a spleen in a patient with functional asplenia. This method is now used to detect the presence of ASs in patients with recurrent chronic idiopathic thrombocytopenic purpura (ITP) after splenectomy (4). It has also been reported in the diagnosis of intrathoracic splenosis (14). Because ^99m^Tc thermally-denatured erythrocytes only accumulate in splenic tissue rather than pNETs, some researchers believe that it can be the best method for diagnosing IPAS (14, 15).

## Conclusion

In conclusion, we believe that CT and MRI still play important roles in the localization and size evaluation of lesions, although they cannot accurately distinguish pNET from IPAS at present. Based on the imaging results in the literature, EUS-FNA should be performed to obtain satisfactory tissue for biopsy. It is suggested that CD8 staining be added on the basis of pNET-related immunohistochemistry to exclude IPAS or avoid unnecessary surgery. Tc-99m heat-denatured red blood cell single photon emission computed tomography is a promising method for diagnosing IPAS, but the number of hospitals performing this technique is relatively limited at present. Based on the factors above, we believe that CT/MRI combined with EUS-FNA can contribute to improving the accuracy of IPAS diagnosis and avoiding unnecessary surgery.

## Data availability statement

The original contributions presented in the study are included in the article/**Supplementary Material**. Further inquiries can be directed to the corresponding authors.

## Ethics statement

Written informed consent was obtained from the individual(s) for the publication of any potentially identifiable images or data included in this article.

## Author contributions

ZYH, SGD and MLD conceived the case and wrote the manuscript. WJ reviewed pathological results. HQQ interpreted the PET/CT results. ZK, DX and LZP interpreted CT results. WJL and JKR helped revise and polish the manuscript. All the authors contributed to the article and approved the submitted version.

## Funding

Our work was supported by the National Science Foundation for Young Scientists of China (No. 81902455); the National Natural Science Foundation of China (No. 82072706, No. 81871980); Jiangsu key Medical Discipline (General Surgery, ZDXKA2016005).

## Acknowledgments

All the authors thank the members of their research group for useful discussions.

## Conflict of interest

The authors declare that the research was conducted in the absence of any commercial or financial relationships that could be construed as a potential conflict of interest.

## Publisher’s note

All claims expressed in this article are solely those of the authors and do not necessarily represent those of their affiliated organizations, or those of the publisher, the editors and the reviewers. Any product that may be evaluated in this article, or claim that may be made by its manufacturer, is not guaranteed or endorsed by the publisher.

## Supplementary material

Supplementary Figure 1 68Ga PET/CT shows an accessory spleen (blue arrow) with low uptake near the spleen. **(A)**. PET image. **(B)**. CT image. **(C)** Fused PET/CT image. **(D)**. Whole-body PET image.

Supplementary Figure 2 68Ga PET/CT shows an accessory spleen (blue arrow) with low uptake near the splenic hilum and tail of the pancreas. **A)**. PET image. **(B)**. CT image. **(C)** Fused PET/CT image. **(D)**. Whole-body PET image.

Supplementary Figure 3 Dual-energy CT examination of the pancreas shows an accessory spleen near the splenic hilum (blue arrow). **(A).** Plain-scan phase. **(B)**. Arterial phase. **(C)**. Venous phase. **(D)**. Delayed phase.

Supplementary Figure 4 Pathological section and immunohistochemical results for SSTR2 and SSTR5 (40X).
