# Supplementary figures and images for: Laparoscopic treatment for an intrapancreatic accessory spleen: A case report
Source: Front Oncol. 2022 Oct 5;12:972883. doi: 10.3389/fonc.2022.972883 (PMC9581263; doi:10.3389/fonc.2022.972883)

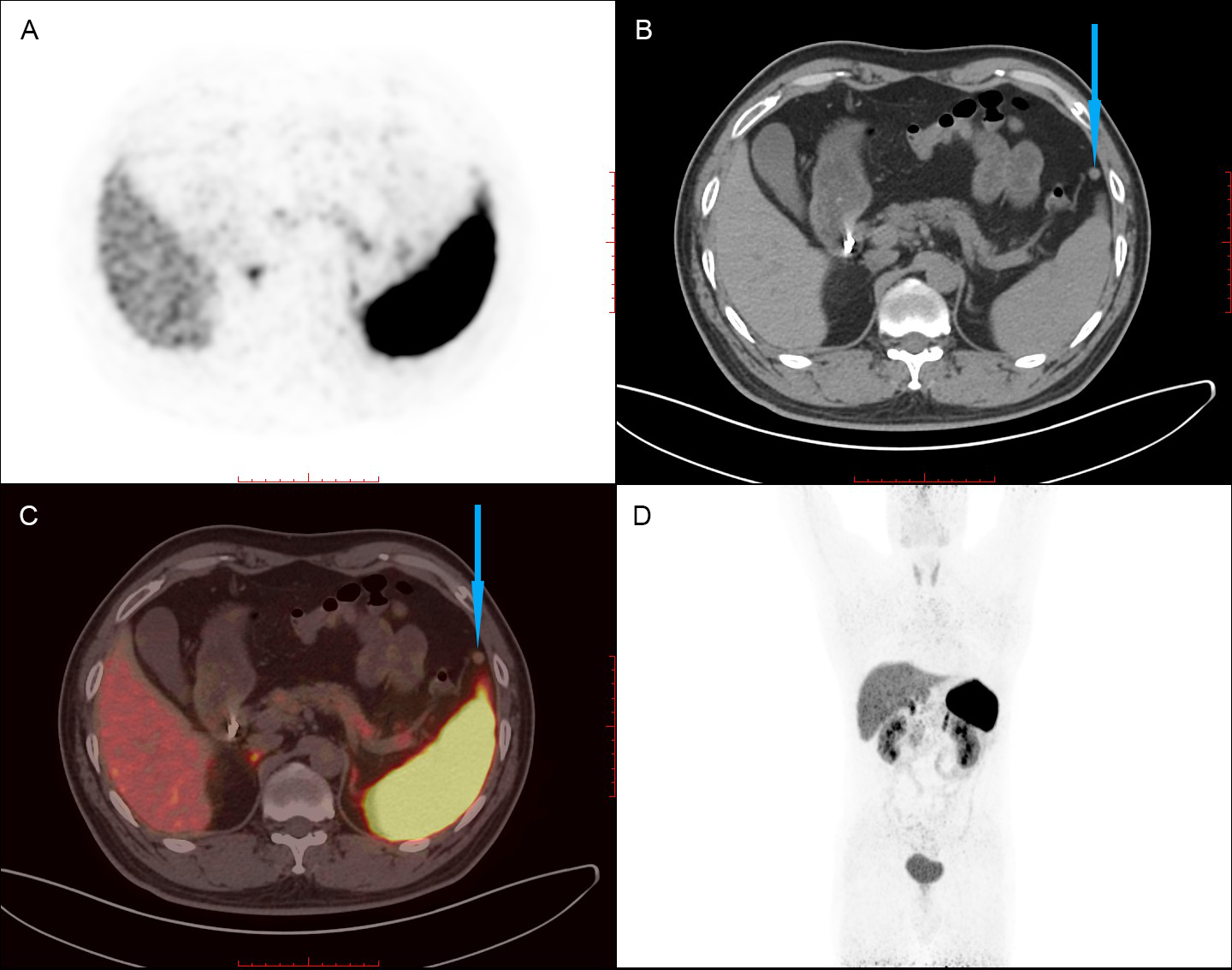

Supplement: Supplementary Figure 1 — 68Ga PET/CT shows an accessory spleen (blue arrow) with low uptake near the spleen. (A). PET image. (B). CT image. (C) Fused PET/CT image. (D). Whole-body PET image. [file Image_1.tif]

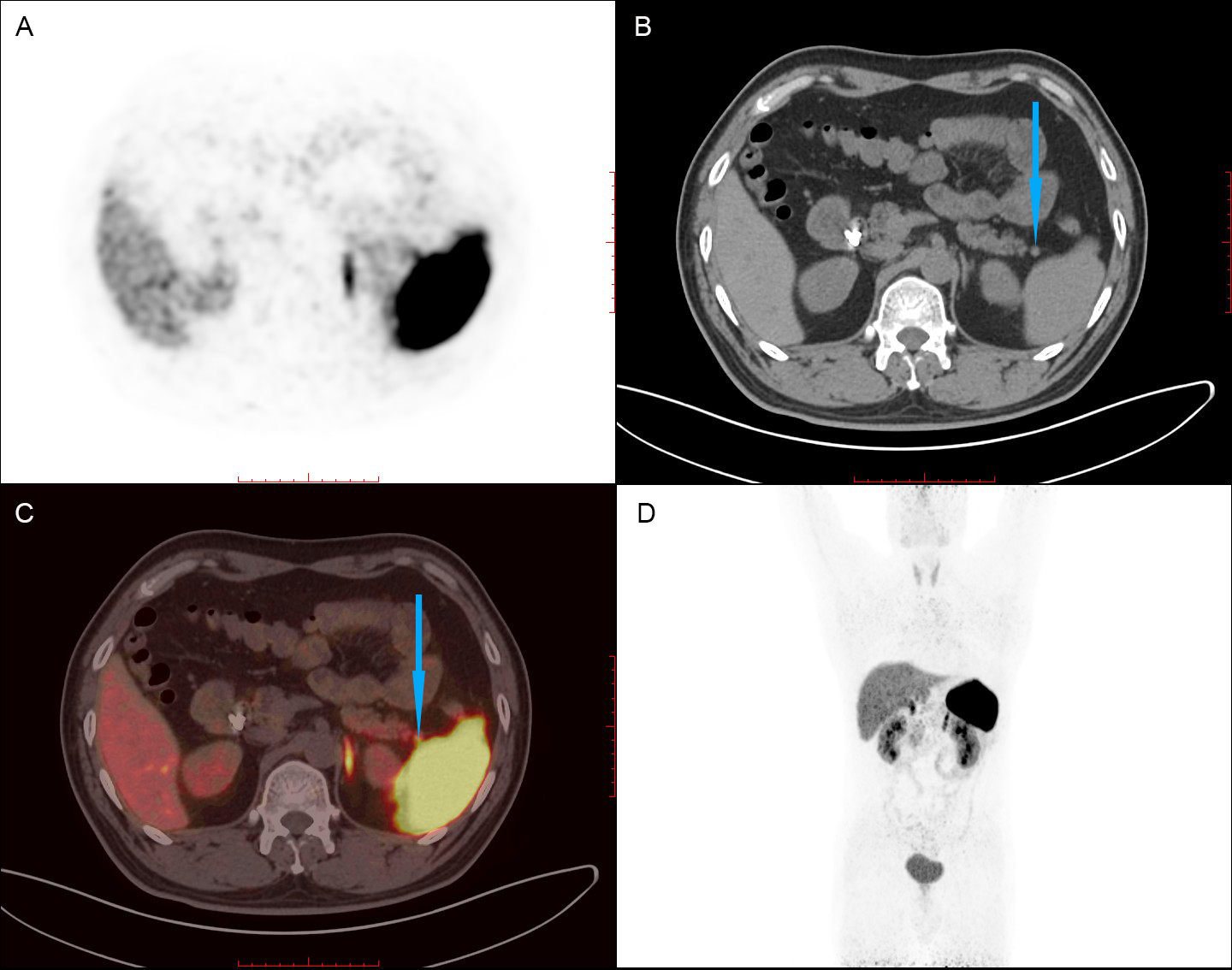

Supplement: Supplementary Figure 2 — 68Ga PET/CT shows an accessory spleen (blue arrow) with low uptake near the splenic hilum and tail of the pancreas. A). PET image. (B). CT image. (C) Fused PET/CT image. (D). Whole-body PET image. [file Image_2.tif]

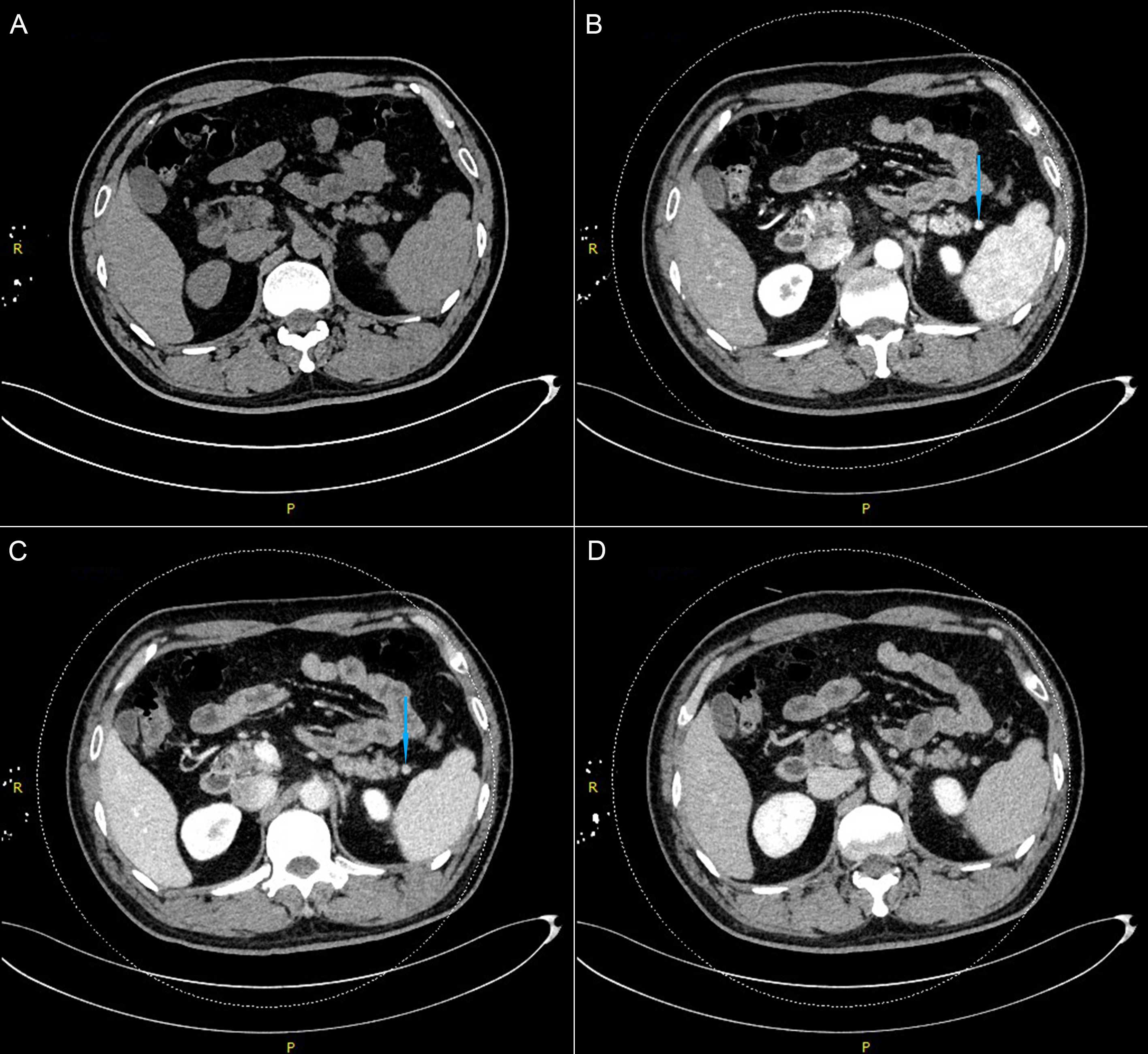

Supplement: Supplementary Figure 3 — Dual-energy CT examination of the pancreas shows an accessory spleen near the splenic hilum (blue arrow). (A). Plain-scan phase. (B). Arterial phase. (C). Venous phase. (D). Delayed phase. [file Image_3.tif]

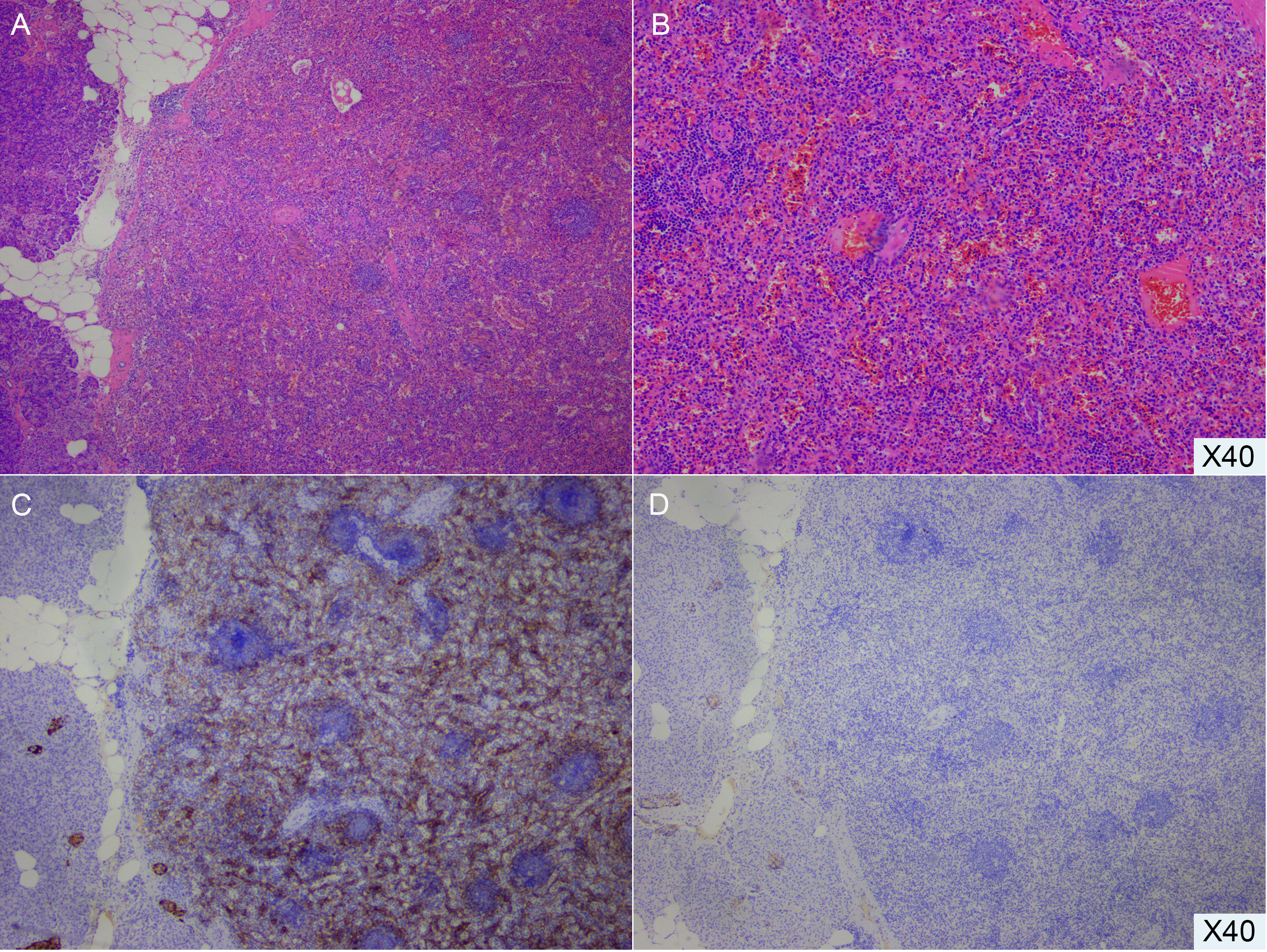

Supplement: Supplementary Figure 4 — Pathological section and immunohistochemical results for SSTR2 and SSTR5 (40X). [file Image_4.tif]
